# Lung disease in crisponi/cold-induced sweating syndrome type 1: expanding the clinical phenotype spectrum

**DOI:** 10.1007/s00431-026-07178-w

**Published:** 2026-10-06

**Authors:** Roberta Onesimo, Cristina De Rose, Nadine Imporporato, Valentina Trevisan, Chiara Leoni, Donato Rigante, Valentina Giorgio, Elisabetta Sforza, Lucrezia Perri, Paola Concolino, Giangiorgio Crisponi, Laura Crisponi, Giuseppe Zampino

**Affiliations:** 1 Center for Rare Diseases, Pediatric Unit, Department of Woman and Child, Fondazione Policlinico Universitario “A. Gemelli” IRCCS, Rome, Italy; 2 Università Cattolica del Sacro Cuore, Rome, Italy; 3 Dipartimento Di Scienze Di Laboratorio Ed Ematologiche, UOC ChimicaBiochimica E Biologia Molecolare Clinica, Fondazione Policlinico Universitario Agostino Gemelli IRCCS, Rome, Italy; 4 Clinica Sant’Anna, Cagliari, Italy; 5 Istituto Di Ricerca Genetica E Biomedica, Consiglio Nazionale Delle Ricerche, Cagliari, Italy; 6 Department of Biomedical Science, University of Sassari, Sassari, Italy

**Keywords:** Aspiration pneumonia, Crisponi syndrome, Lung ultrasound, Personalized medicine, Rare disease, Respiratory difficulties

## Abstract

Crisponi/Cold-Induced Sweating Syndrome Type 1 (CS/CISS1) is an ultra-rare genetic disorder caused by biallelic variants in the cytokine receptor-like factor-1 (*CRLF1*) gene and is associated with several comorbidities. Comprehensive data on the respiratory phenotype of this condition are lacking despite being associated with high mortality in infancy, often due to severe respiratory complications like aspiration pneumonia and sudden death related to hyperthermic crises. Our study investigates respiratory complications and lung ultrasound characteristics, enhancing the comprehension of the natural history and pathophysiology of the disease. We enrolled 13 individuals (8 children) carrying a biallelic molecular defect of the *CRLF1* gene, either in a homozygous or compound heterozygous state. All were of Italian ancestry, with the majority (77%) being Sardinian. The pathogenic variant c.676dup (p.Thr226fs) in exon 4 was confirmed as the most commonly recurrent variant and was identified in 9 of the 13 patients (69%) of our cohort. Over half of the participants exhibited an ultrasound lung pattern suggestive of sonographic interstitial syndrome findings. Fifteen percent had a documented aspiration pneumonia in infancy, which required hospitalization or ventilatory support. Additionally, one adult patient showed bilateral fibrotic findings, which were validated by a CT scan. *Conclusions*: Respiratory involvement appears to be a frequent and under-investigated feature in patients with CS/CISS1. Although lung ultrasound patterns observed in this study were largely nonspecific, LUS may represent a practical, non-invasive screening tool to identify early morphological lung changes potentially related to recurrent or chronic inflammatory processes.

**Table:** 

**What is Known:** •* Crisponi/Cold-Induced Sweating Syndrome Type 1 (CS/CISS1) is associated with early-life respiratory complications.* • *Data on the long-term pulmonary involvement in CS/CISS1 patients are insufficiently documented.*	
**What is New:** • *This is the first study to systematically evaluate lung ultrasound (LUS) findings in a cohort of CS/CISS1 patients, across pediatric and adult ages.* • *We identified recurrent nonspecific interstitial patterns that suggest chronic inflammatory or fibrotic lung involvement, supporting LUS as a feasible screening tool in disease monitoring.*

**Supplementary Information:**

The online version contains supplementary material available at https://doi.org/10.1007/s00431-026-07178-w.

## Introduction

Crisponi/Cold-Induced Sweating Syndrome Type 1 (CS/CISS1, OMIM #272430) is an ultra-rare autosomal recessive genetic disorder described for the first time in 1996 by Giangiorgio Crisponi [1]: in the neonatal period, it is mainly characterized by hyperthermia, camptodactyly, respiratory, and feeding difficulties [1–5] and evolves into a chronic autonomic dysfunction in surviving patients (Buers et al. 2020).

Biallelic pathogenic variants in *CRLF1* or Cardiotrophin-Like Cytokine Factor 1 (*CLCF1*) are associated respectively with CS/CISS type I and type II [2, 5–7]. These genes encode for the soluble type I cytokine receptor CRLF1 (cytokine receptor-like factor-1) and the Interleukin (IL)−6. CRLF1 and CLCF1 form a heterodimeric complex that binds to the Ciliary Neurotrophic Factor Receptor (CNTFR)α/gp130/Leukemia Inhibitory Factor Receptor (LIFR) receptor triplet, triggering intracellular signaling via the Janus Kinase/Signal Transducers and Activators of Transcription (JAK-STAT) pathway, which regulates cell growth and differentiation in the immune and nervous systems [7]. The expression of CRLF1 has been extensively studied in various districts [8, 9], especially in the nervous system, but also appears to be expressed in the respiratory tract [10]. It is highly expressed in the fetal lung, in lung buds and bronchi in the period of branching morphogenesis (E12.5–E18.5) but not in the adult lung [11, 12]. Kass et al. demonstrated that CRLF1 is expressed in type II alveolar epithelial cells and macrophages, where it acts as an inflammatory stimulus with antifibrotic potential in idiopathic pulmonary fibrosis (IPF) [11].

CS/CISS1 syndrome is often linked to higher mortality rates in the first few months of life due to severe respiratory complications, such as aspiration pneumonia and sudden death associated with hyperthermia crises [1, 7, 12]. Although several clinical problems have been reported in CS/CISS1 [1, 2, 5, 13], there is a lack of comprehensive data on the lung and respiratory patterns of these patients.

This prospective observational study aims to investigate respiratory complications and to describe, for the first time, lung ultrasound (LUS) features in a relatively large cohort of patients affected by CS/CISS1, thereby expanding the understanding of this disorder’s natural history.

## Patients and methods

### Study populations and settings

Patients with a confirmed molecular diagnosis of CS/CISS1 syndrome were prospectively recruited over a 12-month period at the Rare Disease Center, Fondazione Policlinico Agostino Gemelli-IRCCS in Rome, Italy. All clinical and genetics data of the patients were previously reported, and no age limit was set for enrollment [2, 4, 12, 14].

Thirteen patients were enrolled in this study (6 males and 7 females; median age: 17 years; age range: 6–44 years); five were adults (38%). None of the included participants was siblings of other participants.

Clinical and respiratory anamnestic data were collected from all patients. Systematic assessment of respiratory symptoms was performed through structured clinical history collection. Lung ultrasound was the only examination systematically performed across the whole cohort, although at different ages. LUS findings were analyzed in relation to medical history and to risk factors potentially influencing the severity of respiratory complications and the occurrence of feeding difficulties. All study procedures adhered to the Declaration of Helsinki, and patients were included in the study after signed informed consent was secured. The Local Ethical Committee—CET Lazio AREA 3—approved the study (approval n.5518 of 2023) as part of an extensive protocol evaluation concerning disabilities in complex rare diseases.

### Genetics analysis

Next generation sequencing (NGS) technique was performed in all patients to confirm previously identified genetic variants and to more accurately characterize identified deletions. Parental segregation, previously defined by Sanger sequencing, was confirmed by allelic phasing in a whole-exome sequencing (WES) study.

Genomic DNA was isolated from peripheral blood leukocytes via an automatic procedure performed using the QIAmp DNA Mini kit on the Qiacube platform (Qiagen, Milan, Italy) following the manufacturer’s indications. Quality checks of the extracted DNA were obtained using the Qubit dsDNA BR fluorometric assays (Life Technologies, Gaithersburg, MD, USA) for quantitation and the spectrophotometry NanoDrop® ND-1000 (Life Technologies, Gaithersburg, MD, USA) for purity evaluation. WES was performed using the Illumina DNA Prep with Exome 2.5 Enrichment kit (Illumina, San Diego, CA, USA), while sequencing was achieved on the NovaSeq 6000 System NGS platform (Illumina, San Diego, CA, USA). Alignment and variant detection were performed with the DRAGEN version 4.3 bioinformatics pipeline (Illumina, San Diego, CA, USA) with GRCh38 as the human reference genome. Tertiary analysis was performed with Emedgene variant interpretation software (Illumina, San Diego, CA, USA) to detect SNVs, indels, and Copy Number Variations (CNVs). For the *CRLF1* gene, the MANE Select Plus Clinical transcript (NM_004750.5 at the time of writing) was used for variants reporting. Identified variants were interpreted in accordance with the American College of Medical Genetics (ACMG) guidelines and the main mutational and population databases such as ClinVar (https://www.ncbi.nlm.nih.gov/clinvar/, accessed on 12 September 2025), LOVD (https://databases.lovd.nl/shared/genes/CRLF1, accessed on 12 September 2025), and gnomADv4.0 (https://gnomad.broadinstitute.org/, accessed on 12 September 2025) [1].

### Lung ultrasound and respiratory evaluation

Major risk factors associated with lung disease development were evaluated in all patients and summarized in Table 1. LUS methodology used was consistent with that implemented for other studies [14, 15]. All lung regions were scanned with LUS to assess pleural slip, line regularity, presence of A lines, density and characteristics of B lines or vertical artifacts, pleural effusions, as well as subpleural lung consolidations. The regular movement of the pleura during breathing was defined as pleural sliding, which, in some instances, may be absent or exhibit irregularities in the hyperechogenicity of the pleural line [17].

**Table 1:** Population data and risk factors for respiratory diseases

Patient ID		1	2	3	4	5	6	7
Age		6	6	9	10	11	11	13
Gender		F	M	M	M	M	F	F
Smoke		-	-	-	-	-	-	-
Aspitarion pneumonia		-	+	-	-	-	+	-
Prematurity		-	-	-	+	-	-	-
GERD		-	-	-	+	+	-	+
Hypertonia of neck muscles		+	-	-	-	-	-	+
Micrognathia		+	-	+	+	+	+	-
Apnea		-	-	-	+	-	-	-
O2 supplementation		-	+	-	+	-	+	-
Scoliosis		-	+	+	+	-	+	+
Cyanosis		-	+	+	+	+	+	+
Swallowing/feeding difficulties		+	+	+	+	+	+	+
Contraction of facial muscles		+	+	+	+	+	+	+
Gene	***CRLF1 [NM_004750.4****]*	H	CH	H	H	CH	H	CH
		c.676dup (p.Thr226fs)	Del:chr19:18,699,611–18,707,859; Del:chr19:18,692,435–18,736,646	c.676dup (p.Thr226fs)	c.676dup (p.Thr226fs)	c.221 T > C (p.Leu74Pro); c.676dup (p.Thr226fs)	c.676dup (p.Thr226fs)	c.226 T > G (p.Trp76Gly); c.676dup (p.Thr226fs)

Patient ID		8	9	10	11	12	13	Prevalence (%)
Age		17	19	25	30	30	44	
Gender		F	F	M	F	M	F	
Smoke		-	-	-	-	-	-	0
Aspitarion pneumonia		-	-	-	-	-	-	15
Prematurity		-	+	-	+	-	+	31
GERD		+	+	-	-	-	-	38
Hypertonia of neck muscles		-	-	+	+	+	+	46
Micrognathia		-	-	+	-	-	-	46
Apnea		+	+	+	+	+	+	54
O2 supplementation		-	+	+	+	-	+	54
Scoliosis		+	+	+	+	-	+	77
Cyanosis		-	+	+	+	-	+	77
Swallowing/feeding difficulties		+	+	+	+	+	+	100
Contraction of facial muscles		+	+	+	+	+	+	100
Gene	***CRLF1 [NM_004750.4****]*	H	H	H	CH	CH	CH	
		c.[338A > T; 341 T > C] p.Asn113Ile; Leu114Pro]	c.226 T > G (p.Trp76Gly)	c.676dup (p.Thr226fs)	c.226 T > G (p.Trp76Gly); c.676dup (p.Thr226fs)	c c.935G > A (p.Arg312His); Del:chr19:18,699,611–18,707,859	c.226 T > G (p.Trp76Gly); c.676dup (p.Thr226fs)	

*CH*, compound heterozygous; *Del*, deletion; *H*, homozygous; *Sa*, Sardinian; *Si*, Sicilian; *Ca*, Calabrian

**Fig. 1:**
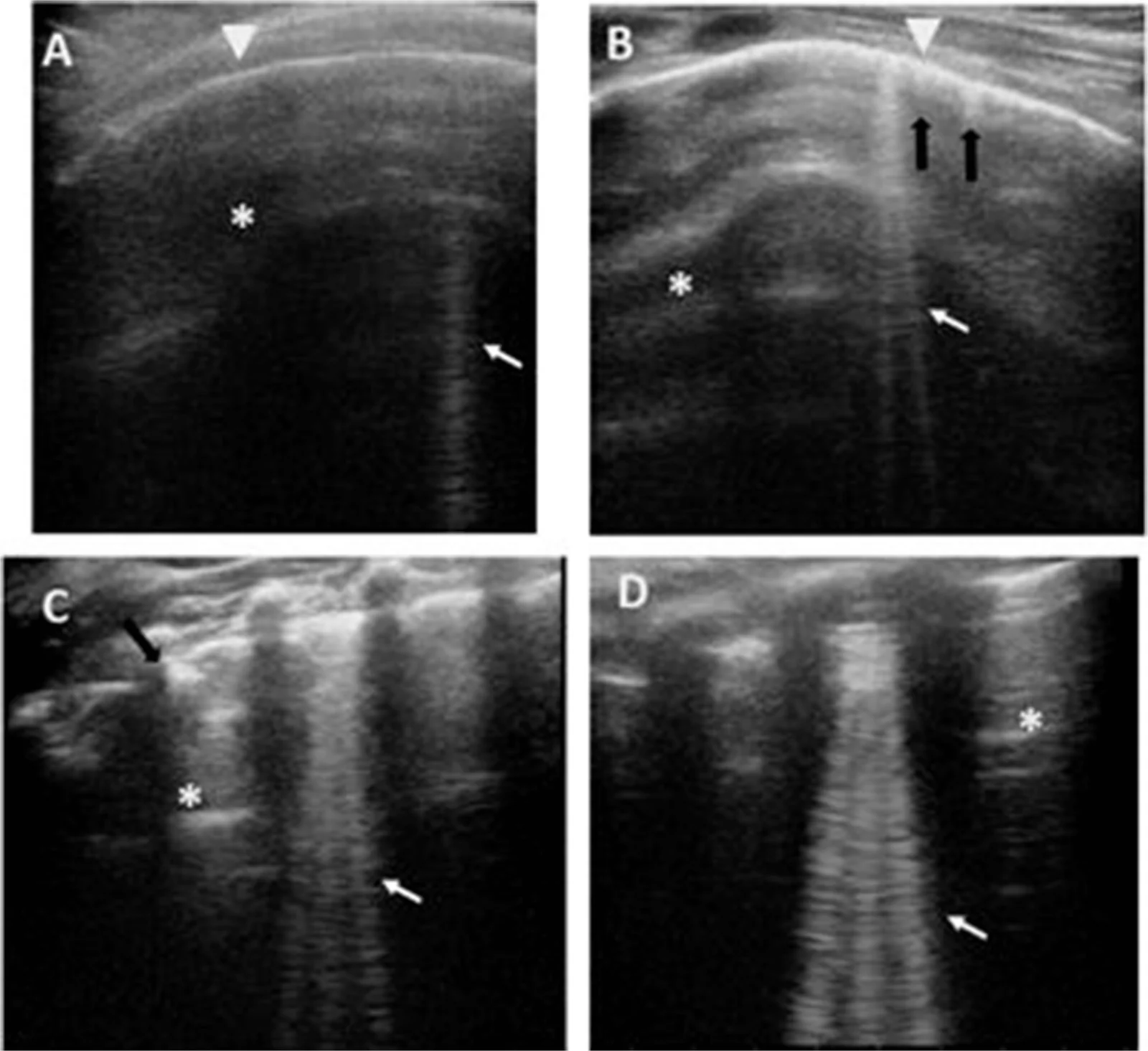
Grayscale lung ultrasound examination (linear probe, 12 MHz, and the small parts preset) shows a SIS picture. The pleural line is regular (arrowheads) with no subpleural consolidations. **B**, **D** Short vertical artifacts (black arrows). **A**, **B** B lines or long vertical artifacts (withe arrows) are single and separated/non-confluent; **C**, **D** they are multiple and confluent. They are distributed unevenly “with spared areas”; where a normal ultrasound pattern with A lines (asterisks) is clearly visible. SIS, sonographic interstitial syndrome

**Fig. 2:**
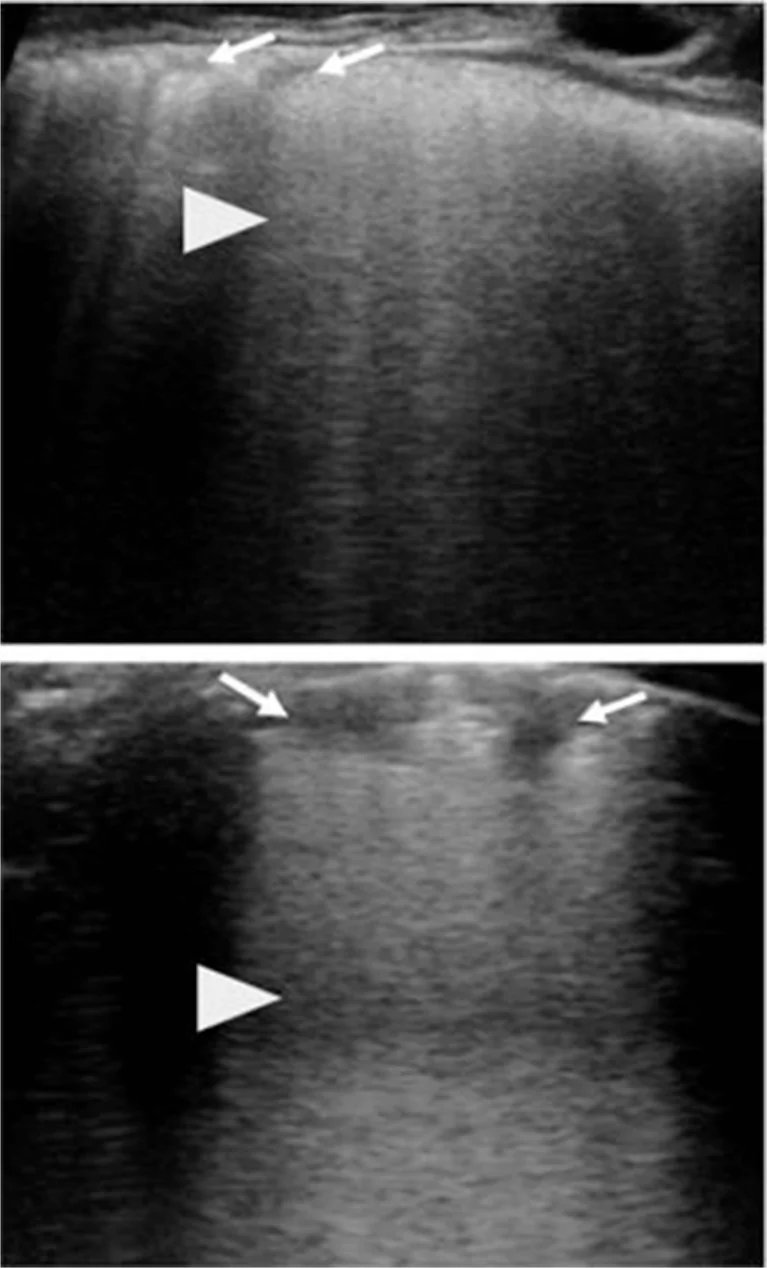
Grayscale lung ultrasound examination (linear probe, 12 MHz, and the small-parts preset) shows a picture of SIS characterized by: the pleural line is irregular. There are small subpleural consolidations (hypoechoic areas, arrows) and below areas of white are evident (arrowheads). B lines or long vertical artifacts are multiple and confluent; they do not have a gravitational trend and do not have a laser artifacts-like appearance; they create areas of white lung (arrowheads) and are unevenly distributed in the explored fields. SIS, sonographic interstitial syndrome

**Fig. 3:**
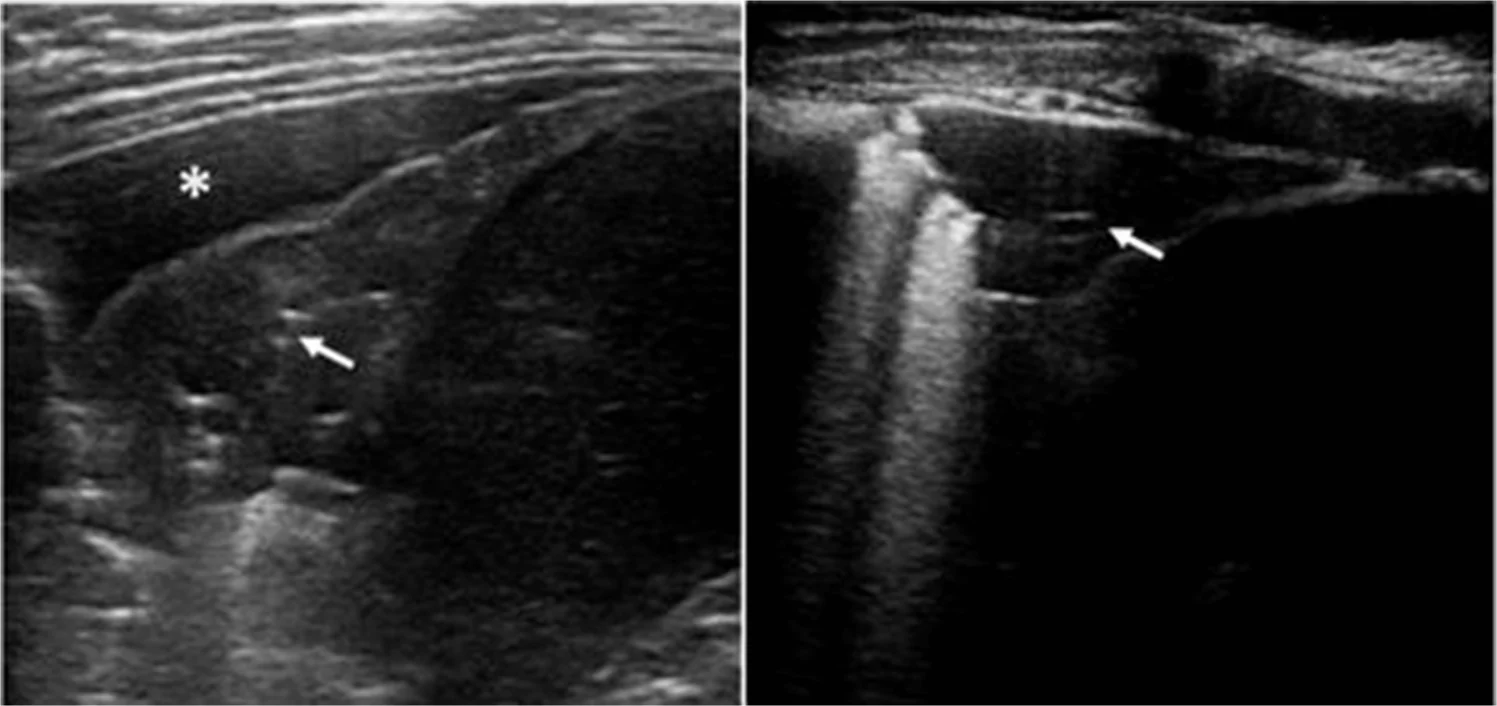
Grayscale lung ultrasound examination (linear probe, 12 MHz, and the small parts preset) shows two atelectatic consolidations characterized by static air bronchograms (arrows). The consolidation in the left figure is associated with reactive pleural effusion (asterisk)

**Fig. 4:**
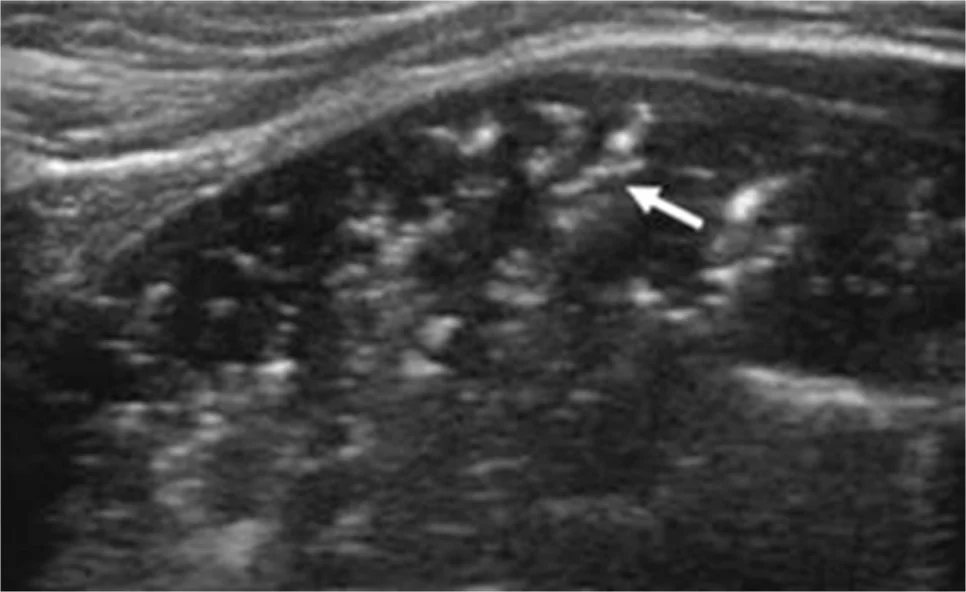
Grayscale lung ultrasound examination (linear probe, 12 MHz, and the small parts preset) shows a consolidation of an inflammatory nature characterized by dynamic air bronchograms (arrows)

The following LUS findings were observed (Figs. 1, 2, 3, and 4):

- Presence of short vertical artifacts
- Presence of long vertical artifacts, their characteristics (multiple or single, confluent or non-confluent), and their position (predominantly localized to the pulmonary bases or widespread in other areas; unilateral or bilateral)
- Presence of anechoic pleural effusion and its location (at the unilateral or bilateral bases)
- Presence of subpleural consolidation and its inflammatory and/or atelectatic characteristics
- Features of the pleural line (regular, irregular, and regular with some irregular areas)

### Statistical analysis

Descriptive statistics were conducted on both demographic and clinical characteristics in the dataset. Results are expressed as percentages.

## Results

Clinical and respiratory data are presented in Table 1.

### Clinical data

Four participants were born preterm (*n* = 4/13, 31%), three of whom were late-preterm. None had a history of bronchopulmonary dysplasia (BPD).

The entire cohort presented feeding difficulties and/or unsafe swallowing at birth, requiring artificial enteral support, as previously reported [16]. Two patients (15%) experienced feeding difficulties until 1 year of age, and two patients (15%) until preschool age. We observed that two patients (15%), aged 10 and 19, respectively, still had persistent eating difficulties, which were evident during our current clinical and ultrasound evaluation. Moreover, five patients reported a history of gastroesophageal reflux disease (GERD) that required medications.

In our cohort, 15% (*n* = 2/13) had documented aspiration pneumonia during the neonatal period, necessitating hospitalization and ventilatory support. The entire cohort had no additional hospitalizations for pneumonias.

Eight patients (*n* = 8/13, 61%) required supplemental oxygen therapy, in addition to their clinical histories and pneumonia episodes. In the early weeks of life, four patients (*n* = 4/13, 31%) needed oxygen, one patient (*n* = 1/13, 8%) continued requiring oxygen until the age of ten, and two patients (*n* = 2/14, 14%) still currently use oxygen when necessary. These two individuals specifically needed oxygen therapy during febrile episodes, which were associated with facial muscle contractions. All patients in the cohort (*n* = 13/13) displayed facial muscle contractions, particularly in early childhood; however, only three patients (*n* = 3/13, 23%) showed this symptom at ages 6 and 11 years.

In total, 77% of our patient cohort (*n* = 10/13) experienced episodes of cyanosis, primarily occurring at birth and resolving within the first few months of life. Among these, two patients continued to have cyanosis until school age, with one experiencing these episodes during febrile episodes. Furthermore, during our clinical and ultrasound assessments, one patient (1/13 = 7%) aged 19 years had persistent acrocyanosis. This evidence indicates that both cyanosis and feeding difficulties improve with age and growth.

Focusing on the ten patients with cyanosis, we observed that five had a history of apnea. Specifically, 54% of the cohort (*n* = 7/13) had a history of sleep apnea. Of these, 21% exhibited this symptom only at birth, while four patients (*n* = 4/13, 31%) aged 11 to 31 years continue to experience sleep disorders characterized by oral breathing, snoring, and apnea. Among patients with a history of apnea, four underwent overnight polysomnographic evaluation when clinically indicated. Notably, only one of these patients reported that their apnea began concurrently with febrile episodes.

We analyzed another clinical factor potentially linked to respiratory issues: the presence of scoliosis, which was found in 85% (*n* = 11/13) of our cohort. This includes two patients (*n* = 2/13, 15%) who underwent corrective orthopedic surgery.

### Genetics data

All subjects were of Italian ancestry, the majority of whom were Sardinian (*n* = 10/13, 77.1%), and were confirmed to carry pathogenic variants or deletions in the *CRLF1* gene in either a homozygous or compound heterozygous state (Table 1). In-depth characterization of the genomics alterations is provided in Supplementary Table 1 and 2. The pathogenic variant c.676dup (p.Thr226fs) was confirmed in 9 out of 13 patients (69%) of our cohort. In five cases, it was present in a homozygous state, and in four, in a compound heterozygous state either with the c.221 T > C (p.Leu74Pro) or with the c.226 T > G (p.Trp76Gly) variant. A patient of Calabrian origin was found to be homozygous for the c.338A > T (p.Asn113Ile) and c.341 T > C (p.Leu114Pro) variants, which were present *in cis*. Pathogenic deletions involving the *CRLF1* gene were identified in two patients of Sicilian origin. In one case, a compound heterozygous state was observed, consisting of an 8.25 kb deletion encompassing *CRLF1* exons from 5 to 9 and the whole *REX1BD* gene, in trans with a larger 44 kb deletion spanning the entire *CRLF1* locus, as well as *TMEM59L* and *REX1B*. In a second individual, a similar deletion (8.25 kb) was detected along with the c.935G > A (p.Arg312His) variant.

### Instrumental and specialistic investigations

Among the instrumental assessments conducted, one patient (*n* = 1/13, 8%) received for clinical issue a contrast chest computed tomography (CT) which revealed bilateral fibrosis, with the right side being more affected than the left one.

Additionally, two patients received ear-nose-throat (ENT) evaluations. In one adult (aged 44 years), the evaluation showed partial paralysis of the left hemilarynx, which led to restricted airflow and difficulty triggering the cough reflex. The other patient (aged 30 years) underwent Functional Endoscopic Sinus Surgery (FESS) due to rhino-sinusal polyposis, but did not experience any improvement of respiratory symptoms, including apnea and nighttime snoring.

We had access to only one pulmonary function test (*n* = 1/13, 8%) from the patient’s clinical data, which indicates a restrictive defect likely linked to scoliosis observed in this patient.

### Lung ultrasound (LUS) data

Ultrasound data are presented in Table 2.

**Table 2:** Lung ultrasound patterns in our cohort of 13 individuals with CS/CISS1

Patient ID		1	2	3	4	5	6	7	8	9	10	11	12	13	Prevalence (%)
Age		6	6	9	10	11	11	13	17	19	25	30	30	44	
Gender		F	M	M	M	M	F	F	F	F	M	F	M	F	
Lung ultrasound	Pleural effusion	-	-	-	-	-	-	-	-	-	-	-	-	-	0
	Bilateral pleural effusion	-	-	-	-	-	-	-	-	-	-	-	-	-	0
	Single B lines	+	-	+	-	-	-	+	-	-	-	-	-	-	23
	Atelectasic consolidation	-	-	-	+	-	-	-	+	+	-	-	-	-	23
	Inflammatory consolidation	-	-	-	+	-	-	-	-	+	-	-	-	+	23
	Multiple not-converging B lines	+	-	-	-	-	-	+	-	-	-	+	-	-	23
	Subpleural consolidation	-	-	-	+	-	-	-	+	+	-	-	-	+	31
	Multiple converging B lines/white lung	-	+	+	+	-	-	-	+	+	-	-	-	+	46
	Multiple B lines	-	+	-	+	-	-	-	+	+	-	+	-	+	46
	Bilateral B lines	+	+	-	+	-	-	-	+	+	-	+	-	+	54
	Irregular pleural line	+	+	+	+	+	+	+	+	+	-	+	-	+	85
	Short B lines	+	+	+	+	+	+	+	+	+	-	+	-	+	85

Legend *M*, male; *F*, female; +, present; −, absent

Most patients (*n* = 11/13, 85%) exhibited an irregular pleural line and short vertical artifacts. In four patients (*n* = 4/13, 31%), lung subpleural consolidations were observed: one patient displayed atelectatic features (*n* = 1/4, 25%), another inflammatory features (*n* = 1/4, 25%), while two patients exhibited both features (*n* = 2/4, 50%).

Two patients (*n* = 2/13, 15%) showed subpleural consolidations in the left posterior basal region. Additionally, one patient (*n* = 1/13, 8%) demonstrated subpleural consolidation in the posterior apical fields on both sides, while another one (*n* = 1/13, 8%) had it in the right paravertebral area. Three patients (*n* = 3/13, 23%) exhibited multiple non-confluent B lines/long vertical artifacts.

In six patients (*n* = 6/13, 46%), multiple confluent long vertical artifacts (white lung) were observed. Four of these patients also exhibited consolidations.

Sixty-one percent (*n* = 8/13) exhibited bilateral confluent long vertical artifacts (white lung): 23% (*n* = 3/13) showed these findings in the right posterior apical and left posterior basal fields; 15% (*n* = 2/13) displayed them in the bilateral posterior basal field; and 15% (*n* = 2/13) revealed bilateral paravertebral confluent vertical artifacts. No patients had pleural effusions on ultrasound.

The LUS image of a 44-year-old female patient displayed multiple regions of white lung/confluent vertical artifacts across both lung fields, along with two consolidations in the right posterior mid-apical area (Figs. 2 and 3). For clinical evaluation, the patient had a chest CT, which revealed bilateral fibrotic changes mainly in the right lung area.

Our evaluation revealed that only two patients (15%), aged 10 and 19 years, respectively, experienced eating difficulties, which persisted during our clinical and ultrasound assessments. Ultrasound findings indicated that both patients exhibited an irregular pleural line and subpleural consolidation with inflammatory and atelectatic features. Additionally, multiple long and confluent vertical artifacts were observed, even in the absence of consolidations in the posterior pulmonary quadrants on both sides (Figs. 2, 3, and 4). Furthermore, in our cohort, 50% of patients with the variant c.226 T > G had consolidations and multiple converging and not B lines, and 75% of them had multiple and bilateral B lines.

## Discussion

CS/CISS1 is an ultra-rare condition which frequently results in respiratory issues [1, 2, 6, 7, 12]: this syndrome is commonly linked to high mortality rates during the initial months of life, primarily due to severe respiratory complications, such as aspiration pneumonia and higher risk of sudden death associated with hyperthermic crises. Our analysis highlights the critical role of a multidisciplinary strategy in addressing respiratory challenges linked to CS/CISS1.

While feeding issues in CS/CISS1 have been well-documented [16], a comprehensive study of the syndrome’s respiratory features has not yet been conducted, leaving a gap in understanding respiratory problems.

In our observational study, we assessed 13 CS/CISS1 patients diagnosed molecularly. For the first time, we collected respiratory data and performed a lung morphological assessment, using LUS, of each individual.

Systematic assessment of respiratory symptoms was performed through structured clinical history collection. However, data collected were not uniform across all patients due to the variability of clinical conditions; for the same reason, instrumental respiratory investigations were not consistently available across the cohort. Pulmonary function testing, chest CT, and overnight polysomnography were performed only in selected cases when clinically indicated. Data on feeding difficulties were also gathered from available medical reports.

Lung ultrasound was the only examination systematically performed across the whole cohort, although at different ages. LUS findings were analyzed in relation to medical history and to risk factors potentially influencing the severity of respiratory complications and the occurrence of feeding difficulties.

Our data indicate that most patients exhibited apnea, cyanosis, feeding difficulties, GERD, and kyphoscoliosis, which could be associated with the presence of respiratory problems. As documented in the literature [1, 2, 6, 7, 12] and confirmed by this study, these symptoms may be less prevalent in adolescence and adulthood. This improvement may be associated with respiratory compensation during development, a hypothesis further supported by the lung ultrasound images we analyzed, which often reveal clinically non-significant features. In addition, while CRLF1 expression in the adult lung is very low or undetectable, its presence during fetal development as seen in the mouse may reflect transient roles in lung maturation. This aligns with its known function as part of the CLCF1–CRLF1 cytokine complex, which signals through the CNTFRα/gp130/LIFR receptor system—a pathway involved in cell survival and differentiation.

*LUS* is a non-invasive, nonionizing radiation tool that provides a rapid, affordable, point-of-care imaging modality for the real-time diagnosis and monitoring of respiratory diseases [15, 17–32], and for some respiratory diseases, it has proven to be more sensitive and specific in the diagnosis and monitoring of pulmonary findings than other methods [19, 26, 28, 30]. Moreover, several studies have identified and validated LUS patterns or scores, mainly based on pleural line irregularities, thickening, vertical artifacts, and subpleural consolidations, to analyze neonatal respiratory conditions [26] or children with disabilities [29, 30, 32]. The latter group and other patient categories exhibit sonographic interstitial syndrome (SIS), characterized by multiple or focal, patched, or diffuse vertical artifacts (B lines) radiating from the lung wall interface, thickening or irregularity of the pleural line, and subpleural consolidations linked to these vertical artifacts/B lines [17]. This LUS pattern is commonly associated with various respiratory diseases [21, 22, 25, 28–30, 33], and, as described in the literature, thanks to the analysis of the characteristics and distribution of the SIS, it is possible to characterize it for each respiratory disease without ever ignoring the clinical, laboratory, functional, and instrumental context.

Eighty-five percent of our patients exhibited ultrasound images with irregularities in the pleural line and unevenly distributed short vertical artifacts between the lung fields. About half of the patients showed multiple and confluent B lines or white lung, which were also unevenly distributed and associated with pleural line irregularities and, in some cases, small subpleural consolidations. These findings are associated with SIS.

Considering the clinical history of these patients, we can infer that ultrasound characteristics observed may suggest a chronic or recurring lung inflammatory process: we wonder whether these observations might indicate signs of interstitial fibrosis, given the presence of CRLF1 in type II alveolar epithelial cells and macrophages. Studies have shown that this expression acts as an inflammatory stimulus with antifibrotic properties in IPF [7, 11]. To accurately explain and validate these data and our hypothesis, a larger patient sample, along with supporting respiratory function tests and advanced imaging studies, will be necessary. Conversely, the ultrasound features identified may correlate with the results of recurrent micro aspirations, typical in patients with history of eating or swallowing difficulties [34–42].

Among our cohort, 15% of patients (*n* = 2/13) were diagnosed with aspiration pneumonia at birth and required hospitalization as well as ventilatory support. The LUS results for these three patients showed an irregular pleural line and short vertical artifacts, although only one exhibited an area of white lung. None of the patients displayed consolidations on LUS.

By the first months of life, most patients no longer had feeding/swallowing difficulties. Our data indicate that this improvement in swallowing function corresponds with morphological compensation, as suggested by our ultrasound results, which reveal mild abnormalities in most patients.

We observed that only two patients (15%), aged 10 and 19 years, experienced eating difficulties, which were still evident during our current clinical and ultrasound assessments. Regarding the ultrasound findings, both patients displayed an irregular pleural line and small subpleural consolidations that exhibited both inflammatory and atelectatic characteristics. These were associated with numerous long and confluent vertical artifacts, visible even when no consolidation was detected in the bilateral posterior lung fields (Figs. 3 and 4). This pattern may indicate chronic inflammatory changes resulting from repeated pulmonary microaspirations; additionally, this ultrasound pattern is nonspecific and should be considered in differential diagnoses alongside other causes of interstitial syndromes.

Among the six patients showing white lung and consolidations on ultrasound evaluation, 50% (*n* = 3/6) were born late-preterm and had low birth weight. At the time of our clinical evaluation, two of these patients were adults.

Most patients (*n* = 5/6, 83%) exhibited cyanosis and apnea at birth. During clinical and ultrasound assessments, two patients (*n* = 2/6, 33%) continued to show cyanosis, while three (*n* = 3/6, 50%) still experienced apnea at the time of our evaluation.

All six patients in the study cohort displaying this ultrasound pattern had a history of scoliosis, with two having previously undergone orthopedic surgery. Moreover, five patients reported a previous history of gastroesophageal reflux disease that required medication. Among these patients, a 17-year-old girl was diagnosed with evidence of gastritis by *Helicobacter pylori* at the esophagogastroduodenoscopy, and a 19-year-old girl with a diagnosis of gastroesophageal reflux disease proven by pH impedenzometry. One patient (20%) continued to exhibit symptoms of gastroesophageal reflux disease during our clinical and ultrasound assessment.

Regarding characterization of the *CRLF1* variant cohort, 9 out of 13 patients—born to apparently unrelated parents—shared the same variant and had Sardinian ancestry. Sardinia represents a classic founder population shaped by prolonged geographic isolation, recurrent demographic bottlenecks, and limited gene flow. These factors increase the frequency of ancestral variants, enrich for founder mutations and rare diseases, and raise the likelihood that unrelated individuals carry the same pathogenic *CRLF1* variant. Therefore, we hypothesize that the observed variants are consistent with a founder effect (PMID: 17,436,252). Moreover, the similar deletion dimension (8.25 Kb) detected in patient 2 and 12 and same geographic origin (Sicilian) might entail a common genetic ancestor or a founder effect.

Furthermore, we attempted to do a genotype/phenotype correlation in those patients exhibiting lung findings and consolidations on LUS. Out of the six patients with the most significant ultrasound characteristics, three patients share the c.676dup in homozygous state, while one subject carried the c.226 T > G variant on the second allele. In contrast, the other two patients carried different variants that are less represented in the population. In detail, a 6-year-old Sicilian boy was found carrying a double deletion in compound heterozygous state and a 17-year-old Calabrian girl was confirmed carrying the c.338 A > T (p.Asn113Ile); 341 T > C (p.Leu114Pro) *in cis* in homozygous state (Table 1).

We further compared the most prevalent respiratory clinical features with the underlying genetic variants, observing that among the nine patients carrying the c.676dup variant, only 33% exhibited a history of cyanosis, apnea, and intermittent oxygen requirement; among these three individuals, only one had a documented history of aspiration pneumonia. A genotype–phenotype correlation was evaluated using the available cohort and clinical data. However, the limited sample size precluded the identification of consistent or statistically robust associations. As a result, observed clinical variability cannot be confidently attributed to specific variants, and further studies involving larger patient populations are necessary to clarify potential associations.

This study has several limitations. The first is the small sample size, which is a consequence of the participants having an extremely rare disease, and this may have influenced our findings. However, we think that the topic cannot be considered too rare or specific, given the importance of rare diseases in the current pediatric panorama. Indeed, while “old common pediatric diseases" have been fought, rare diseases become more and more important as we know their mechanisms and their societal importance is highlighted thanks to family advocacy.

Secondly, the nonspecific nature of the ultrasound findings limits our ability to provide a definitive diagnosis. While LUS may serve as a basic method for detecting lung pattern abnormalities, other diagnostic tools like high resolution computed tomography should be employed to accurately diagnose and classify chronic fibrotic changes and/or inflammatory patterns, which will help standardize the ultrasound results. Additionally, to comprehensively evaluate the respiratory conditions of these patients, in future studies, it is essential to include respiratory function assessments, such as spirometry and polysomnography. Furthermore, the cohort includes only surviving patients, introducing a potential survival bias and limiting the assessment of the role of respiratory complications in mortality.

Expanding the sample size and establishing research networks and collaborations to gather additional data would greatly enhance the clinical significance of our study.

Our preliminary work, which has shown that respiratory involvement is a frequent feature in patients with CS/CISS1, will provide the basis for a future prospective study that involves systematic respiratory assessment of patients with Crisponi syndrome, with the possibility of correlating the new lung ultrasound technique with established functional, clinical, and instrumental standards.

Future studies are needed to understand whether lung ultrasound can be a practical and non-invasive screening tool for this ultra-rare disease.

## Conclusion

Respiratory involvement appears to be a frequent feature in patients with CS/CISS1. Although lung ultrasound patterns observed in this study were largely nonspecific, LUS may represent a practical, non-invasive screening tool to identify early morphological lung changes potentially related to recurrent or chronic inflammatory processes. Given the heterogeneity of respiratory assessment and the methods used mostly relating to the anamnestic collection to define the respiratory patterns of each patient, these findings should be interpreted with caution. Nonetheless, in the context of this ultra-rare syndrome, early respiratory monitoring, including LUS, may improve standard of care, facilitate timely recognition of complications, guide multidisciplinary management, and ultimately increase clinical outcomes and quality of life.

## Supplementary Information

Below is the link to the electronic supplementary material.

Supplementary file1 (DOCX 17 KB)

## Data Availability

The datasets generated during the current study are available from the corresponding author on reasonable request.

## Abbreviations

BPD: Bronchopulmonary dysplasia
CAP: Community acquired pneumonia
CLCF1: Cardiotrophin-Like Cytokine Factor 1
CNTFR: Ciliary Neurotrophic Factor Receptor
CRLF1: Cytokine receptor-like factor-1
CS/CISS1: Crisponi Syndrome/Cold-Induced Sweating Syndrome Type 1
CT: Computed tomography
ENT: Ear-nose-throat
FESS: Functional Endoscopic Sinus Surgery
GERD: Gastroesophageal reflux disease
HRCT: High resolution computed tomography
IL-6: Interleukin 6
IPF: Idiopathic pulmonary fibrosis
JAK-STAT: Janus Kinase/Signal Transducers and Activators of Transcription
LIFR: Leukemia inhibitory factor receptor
LUS: Lung ultrasound
SIS: Sonographic interstitial syndrome
