# Supplementary material for: Lung disease in crisponi/cold-induced sweating syndrome type 1: expanding the clinical phenotype spectrum
Source: Eur J Pediatr. 2026 Oct 6;185(11):805. doi: 10.1007/s00431-026-07178-w (PMC13638677; doi:10.1007/s00431-026-07178-w)
Supplement: Supplementary file 1 [file 431_2026_7178_MOESM1_ESM.docx]

| **CRLF1** | | **Present report** | **ClinVar ID** | **LOVD** | **PMID** |
| --- | --- | --- | --- | --- | --- |
| **Coding NM_004750.5** | **Protein NP_004741.1** | **ACMG** |  |  |  |
| c.221T>C | (p.Leu74Pro) | LP: PM1; PM2; PM3; PP3 | 3375191 | Pathogenic | 24488861 |
| c.226T>G | (p.Trp76Gly) | LP: PM1; PM2; PM5 PP3; PP5 | 5708 | Pathogenic | 24488861; 17436252; 17436251; 8723066 |
| c.338A>T | (p.Asn113Ile) | Hot VUS: PM1; PM2; PP3; PP5 | NA | Pathogenic | 21326283 |
| c.341T>C | (p.Leu114Pro) | Hot VUS: PM1; PM2; PP3; PP5 |  | Likely pathogenic | 21326283 |
| c.676dup | (p.Thr226fs) | P: PVS1; PM2; PP5 | 5707 | Pathogenic | 24488861; 17436252; 17436251; 8723066 |
| c.935G>A | (p.Arg312His) | LP: PM1; PM2; PM3; PP3; PM5 | 4536057 | Pathogenic | 20975058 |

**Supplementary Table 1** Genetic variants identified in our monocentric cohort.

**Supplementary Table 1 Legend:** ACMG American Association of Medical Genetics and Genomics;; LOF: Loss of function; LOVD: Leiden Open Variation Database; LP: Likely Pathogenic; P: Pathogenic; VUS: Variant of Unknown Significance.

**Supplementary Table 2** Molecular deletions identified in our monocentric cohort.

| Molecular rearrangement | Genomic coordinates (GRCh37) | size (Kb) | Specific *CRLF1* exons and contiguous genes involved | Clinical significance (Present report) |
| --- | --- | --- | --- | --- |
| Deletion | 19:18699611-18707859 | 8.25 | from 5 to 9 | LP: Overlap with established HI/LOF-sensitive genes or genomic regions |
| Deletion | 19:18692435-18736646 | 44 | contiguous gene deletion (*CRLF1, TMEM59L, REX1B*) | LP: Overlap with established HI/LOF-sensitive genes or genomic regions |
| Deletion | 19:18699611-18707859 | 8.25 | from 5 to 9 | LP: Overlap with established HI/LOF-sensitive genes or genomic regions |

**Supplementary Table 2 Legend**: GRCh37: Genome Reference Consortium Human Build 3; HI: Haploinsufficiency; Kb: Kilobase; LOF: Loss of function.
